# Supplementary material for: Clinical Evidence of Biomimetic Hydroxyapatite in Oral Care Products for Reducing Dentin Hypersensitivity: An Updated Systematic Review and Meta-Analysis
Source: Biomimetics (Basel). 2023 Jan 6;8(1):23. doi: 10.3390/biomimetics8010023 (PMC9844412; doi:10.3390/biomimetics8010023)
Supplement: Supplementary file 1 [file biomimetics-08-00023-s001.zip › Supplement TableS3-Limeback_DH_Review-HealthCanadaApprovedHAPtoothpastes.pdf]

Table S3. Health Canada Approved Hydroxyapatite Toothpastes

Brand name (Company)	Natural Product Number (date of licensing)	% hydroxyapatite / medicinal ingredients	Other ingredients	Recommended use or purpose / Sub population
X-Pur Remin (Oral Science Inc)	80061255 (2015-05-27) revised (2021-10-18)	10%	Carboxymethylcellulose sodium<br>Carrageenan<br>Cetylpyridinium Chloride<br>Ethanol<br>Glycerin<br>Glycyrrhetinic acid<br>Macrogol 400<br>Peppermint Flavour<br>Polyoxyethylene Castor Oil Derivatives<br>Polyvinylpyrrolidone<br>Silicon dioxide<br>Sodium Lauroyl Glutamate<br>Titanium dioxide<br>Water<br>Xylitol<br>Zeolite	Helps remineralize tooth enamel<br>Helps restore minerals to tooth enamel<br>Strengthen teeth by remineralizing enamel<br>Helps to reduce cavities<br>Helps to reduce tooth sensitivity pain<br>• children 2-5 yrs<br>• children and adolescents 6 – 11 yrs<br>• adults and adolescents >/= 12 yrs
UltraDex Sensitive Toothpaste (Venture Life Ltd.)	80081856 (2017-12-14) revised (2018-02-17)	7.5% (+ 1.09% sodium monofluorophosphate)	Aqua<br>C.I. No. 77891<br>Cellulose gum<br>Citric acid<br>Glycerin<br>Hydrated Silica<br>Menthol<br>Sodium bicarbonate<br>Sodium chlorite<br>Sodium citrate<br>Sodium phosphate tribasic dodecahydrate<br>Sodium saccharin<br>Spearmint essential oil<br>d-Limonene<br>l-Limonene	Helps to rebuild tooth surfaces<br>Provides a protective enamel shield<br>Protects teeth<br>Strengthen teeth by remineralizing enamel<br>Helps rebuild tooth surfaces, to leave teeth stronger<br>• adults
SirinMED Hyper Senstivie Enamel Protection (Bukwang Pharmaceutical Co. Ltd.)	80082864 (2018-02-05)	19% (+ 2.5% silicon dioxide)	Allura Red AC<br>Carboxymethylcellulose sodium<br>Fragrance<br>Glycerin<br>Menthol<br>Methylparaben<br>Purified water<br>Saccharin sodium dihydrate<br>Sodium Cocoyl Isethionate<br>Sodium lauryl sulfate	Provides clinically shown relief from dentin hypersensitivity pain<br>• keep out of children under 6 years of age
Extra Whitening Toothpaste (Green Beaver Co. Ltd.)	80090492 (2019-02-07) revised (2020-11-30)	4.25% (+ 20.0% xylitol)	Aqua<br>Calcium carbonate<br>Carica Papaya (Papaya) Fruit Extract<br>Citrus grandis (grapefruit) seed extract<br>Citrus grandis (grapefruit) seed extract<br>Coco-Glucoside<br>Glycerin<br>Hydrated Silica<br>Maltodextrin<br>Menthol<br>Sorbitol<br>Tea tree essential oil<br>Xanthan Gum	Helps prevent dental cavities<br>Helps prevent plaque formation<br>Helps reduce the risk of tooth decay<br>• adults and children minum age 6 years

Enamel Shield Toothpaste<br>(Green Beaver Co. Ltd.)	80091224<br>(2019-03-14)<br>revised<br>(2020-12-02)	4.25%<br>(+ 20.0% xylitol)	Aqua<br>Calcium carbonate<br>Carica Papaya (Papaya) Fruit Extract<br>Citrus grandis (grapefruit) seed extract<br>Citrus grandis (grapefruit) seed extract<br>Coco-Glucoside<br>Glycerin<br>Hydrated Silica<br>Maltodextrin<br>Menthol<br>Sorbitol<br>Tea tree essential oil<br>Xanthan Gum	Provides a protective enamel shield<br>Helps prevent dental cavities<br>Helps prevent plaque formation<br>Helps reduce the risk of tooth decay<br>• adults and children minimum age 6 years
Apagard Premio<br>(Sangi Co. Ltd.)	80096121<br>(2019-12-16)<br>revised<br>(2021-01-22)	7.0%	Aqua<br>Cellulose gum<br>Cetylpyridinium chloride<br>Dicalcium phosphate<br>Flavour<br>Glycerin<br>Glycyrrhetinic acid<br>Magnesium phosphate, tribasic<br>PEG-8<br>Silica<br>Sodium lauryl sulfate<br>Sodium saccharin<br>Sodium silicate<br>Xylitol	Helps remineralize tooth enamel<br>Helps restore minerals in tooth enamel<br>Strengthens tooth enamel<br>Helps to reduce cavities<br><br>• adolescents 9 to 13 yrs<br>• adults and adolescents 15 years min.<br>• adults and adolescents 12 years min<br>• children 2 to 3 years<br>• children 4 to 9 years
Apadent Total Care<br>(Sangi Co. Ltd.)	80096124<br>(2019-12-16)<br>revised<br>(2021-01-22)	7.0%	Aqua<br>Butylene glycol<br>Cellulose gum<br>Cetylpyridinium chloride<br>Chamomilla Recutita (Matricaria) Flower Extract<br>Dicalcium phosphate<br>Ethanol<br>Flavour<br>Glycerin<br>Green tea extract<br>Magnesium phosphate, tribasic<br>PEG-8<br>PVP<br>Sage Leaf Extract<br>Silica<br>Sodium lauroyl sarcosinate<br>Sodium lauryl sulfate<br>Sodium saccharin	Helps remineralize tooth enamel<br>Helps restore minerals in tooth enamel<br>Strengthens tooth enamel<br>Helps to reduce cavities<br><br>• adolescents 9 to 13 yrs<br>• adults and adolescents 15 years min.<br>• adults and adolescents 12 years min<br>• children 2 to 3 years<br>• children 4 to 9 years
Kinder Karex<br>(Dr. Kurt Wolff GmbH & Co. KG)	80117093<br>(2022-03-12)	10%	1,2-Hexanediol<br>Aqua<br>Caprylyl glycol<br>Cellulose gum<br>Flavour<br>Glycerin<br>Hydrated Silica<br>Hydrogenated Starch Hydrolysate<br>Silica<br>Sodium cocoyl glycinate<br>Sodium methyl cocoyl taurate<br>Sodium sulfate<br>Xylitol	Helps prevent (dental) cavities<br>Helps fight (dental) decay<br>Cavity protection<br>Helps remineralize tooth enamel<br>Helps rebuild tooth enamel<br>Helps reinforce tooth enamel<br>Helps prevent tooth enamel mineral loss<br>Helps protect tooth enamel<br><br>• all ages children, adolescents and adults, min. age 2 years
