# Supplementary material for: Clinical Evidence of Biomimetic Hydroxyapatite in Oral Care Products for Reducing Dentin Hypersensitivity: An Updated Systematic Review and Meta-Analysis
Source: Biomimetics (Basel). 2023 Jan 6;8(1):23. doi: 10.3390/biomimetics8010023 (PMC9844412; doi:10.3390/biomimetics8010023)
Supplement: Supplementary file 1 [file biomimetics-08-00023-s001.zip › Supplement-Limeback_DH_Review-PRISMA-S_Checklist.pdf]

PRISMA-S Checklist- Hydroxyapatite and Dentin Hypersensitivity

Section/topic	#	Checklist item	Location(s) Reported
**INFORMATION SOURCES AND METHODS**			
Database name	1	Name each individual database searched, stating the platform for each.	OVID Medline/PubMed, Elsevier Scopus, EMBASE, Cochrane Library, Web of Science, Google Scholar
Multi-database searching	2	If databases were searched simultaneously on a single platform, state the name of the platform, listing all of the databases searched.	University of Toronto Library
Study registries	3	List any study registries searched.	clinicaltrials.gov/ and cochranelibrary.com/central but unpublished studies were not included in the systematic analysis
Online resources and browsing	4	Describe any online or print source purposefully searched or browsed (e.g., tables of contents, print conference proceedings, web sites), and how this was done.	IADR.org was searched for relevant abstract that would indicate potential publications
Citation searching	5	Indicate whether cited references or citing references were examined, and describe any methods used for locating cited/citing references (e.g., browsing reference lists, using a citation index, setting up email alerts for references citing included studies).	Titles were browsed in all databases. The citations in the reference lists of the full papers retrieved and read and were browsed for citations missed in the literature search.
Contacts	6	Indicate whether additional studies or data were sought by contacting authors, experts,	One expert in the dental profession in Canada was

		manufacturers, or others.	contacted about hydroxyapatite used in dentistry
Other methods	7	Describe any additional information sources or search methods used.	Contact with dental professionals in Germany revealed several foreign language studies (which were identified in the Scopus search)
**SEARCH STRATEGIES**			
Full search strategies	8	Include the search strategies for each database and information source, copied and pasted exactly as run.	See table (Supplement)
Limits and restrictions	9	Specify that no limits were used, or describe any limits or restrictions applied to a search (e.g., date or time period, language, study design) and provide justification for their use.	The final date of the search was May 1, 2022. All languages were accepted. All open access papers were accepted.
Search filters	10	Indicate whether published search filters were used (as originally designed or modified), and if so, cite the filter(s) used.	No filters were used.
Prior work	11	Indicate when search strategies from other literature reviews were adapted or reused for a substantive part or all of the search, citing the previous review(s).	A database was published in a previous systematic review (Limeback et al, 2021, ref. 18) and cross-checked for citations in dentin hypersensitivity
Updates	12	Report the methods used to update the search(es) (e.g., rerunning searches, email alerts).	Two authors had a previous list of published papers. These provided 3 additional publications found outside of the search
Dates of searches	13	For each search strategy, provide the date when the last search occurred.	May 1, 2022 (all searches)

**PEER REVIEW**			
Peer review	14	Describe any search peer review process.	Peer review of the search was not done externally.
**MANAGING RECORDS**			
Total Records	15	Document the total number of records identified from each database and other information sources.	See Supplement table S2.
Deduplication	16	Describe the processes and any software used to deduplicate records from multiple database searches and other information sources.	Duplicates were found manually and by using key word searches in pdf printouts of lists of papers generated in the searches

Rethlefsen ML, Kirtley S, Waffenschmidt S, Ayala AP, Moher D, Page MJ, et al. PRISMA-S Group PRISMA-S: An Extension to the PRISMA Statement for Reporting Literature Searches in Systematic Reviews [17].
