# Supplementary material for: Clinical Evidence of Biomimetic Hydroxyapatite in Oral Care Products for Reducing Dentin Hypersensitivity: An Updated Systematic Review and Meta-Analysis
Source: Biomimetics (Basel). 2023 Jan 6;8(1):23. doi: 10.3390/biomimetics8010023 (PMC9844412; doi:10.3390/biomimetics8010023)
Supplement: Supplementary file 1 [file biomimetics-08-00023-s001.zip › Supplement-TableS1-Limeback_DH_Review-Systematic reviews on DH.pdf]

Supplement Table S1. Summary of recent systematic reviews of dentin hypersensitivity that included hydroxyapatite (HAP)

Study authors	End date of search	Databases searched	Total RCTs included	Active ingredients investigated	RCTs of in-office treat-ments included ?	RCTs of treating DH during vital bleaching included?	RCTs of DH after perio-dontal therapy included ?	Meta-analysis reported ?	Number of HAP trials included	Authors' Conclusions
Mora-schini et al., 2018 [8]	Mar. 2017	Medline (PubMed), Cochrane Library	21	Laser, fluoride (gel, varnish, DSF), potassium, DSP, arginine, n-HAP	yes	yes	yes	yes	3	"There was no significant difference in the direct comparison between the treatment groups. For in-home treatments, only chemical occlusion of dentin tubules and nerve desensitization showed a greater treatment efficacy than placebo, and the difference was statistically significant."
Hu et al., 2018 [9]	Nov. 2017	PubMed, EMBASE, Web of Science, Cochrane CENTRAL, Chinese Biomedical Literature Database	53	KNO_3, KCl, $SrCl_2$, SnF_2, CSPS, Arginine/$CaCO_3$, ACP, n-HAP	no	no	no	yes	2	"Desensitizing toothpastes containing potassium, stannous fluoride, potassium and strontium, potassium and stannous fluoride, calcium sodium phosphosilicate, arginine, and nano-hydroxyapatite are effective in relieving DH"
Gul et al., 2021	2018	PubMed, Science Direct, Web of Science	16 (in-cludes in vitro	HAP compared to positive controls (NovaMin,	no	no	no	no	7	"Clinical trials (…) reported reduction in DH in patients following 2-8 weeks of

[10]			studies)	ProArgin, KNO_3)						usage.”
De Melo Alencar et al., 2019<br><br>[11]	Apr. 2018	PubMed, Web of Science, Cochrane CENTRAL, Scopus, LILACS, ClinTrials.gov, OpenGrey	8	n-HAP compared to positive controls	no	no	no	yes	8	“The n-HAP-containing treatment showed better clinical performance than other treatments for DH relief.”
Marto et al., 2019<br><br>[12]	Nov. 2018	Medline/PubMed EMBASE, Cochrane Library, ClinicalTrials	66	Potassium, fluoride, strontium, oxalates, iontophoresis, arginine, glutaralde-hyde + HEMA, chlorhexidine, HAP, herbal, ozone, composite resins, adhesives, GIC, sealants, laser	yes	yes	yes	no	20	“For long-time effects, at-home treatments with chemical agents, such as potassium nitrate, arginine or hydroxyapatite, can also be used to treat DH with significant results.”
Hu et al., 2019<br><br>[13]	Dec. 2018	Medline, EMBASE,<br><br>Web of Science, Cochrane CENTRAL	30	KNO_3, KCl, $SrCl_2$, SnF_2, CSPS, Arginine/ $CaCO_3$, ACP, n-HAP	no	no	no	yes<br><br>(Net-work)	2	“n-HA containing toothpastes may be the best desensitizing toothpaste for the treatment of DH.”
Martins et al., 2020<br><br>[14]	Feb. 2019	Medline, EMBASE, Cochrane CENTRAL	90	>300 comparisons involving fluoride, CSP, SnF_2, TCP, arginine, $SrCl_2$, Herbal, KNO_3, n-HAP	no	no	no	yes<br><br>(Networ k)	1	“Most toothpaste formulations showed evidence of superiority against placebo or fluorides (amine fluoride, MFP, or NaF) alone in managing all forms of DH (high to moderate certainty). Strontium and potassium showed moder- ate

										effectiveness for tactile stimulus and arginine for air stimulus. The combination of potassium with SnF_2 or hydroxyapatite was effective for tactile and air stimuli with moderate certainty of evidence.”
Ouben-yahya, 2021<br><br>[15]	May. 2021		7 (double blinded RCTs)	HAP vs placebo or positive controls	no	no	no	no	7	“No conclusion of superiority of n-HA can be ascertained when compared to other desensitizing molecules (…) n-HA remains an effective desensitizing agent to consider as a therapeutic option in everyday practice.”
