# Supplementary material for: Clinical Evidence of Biomimetic Hydroxyapatite in Oral Care Products for Reducing Dentin Hypersensitivity: An Updated Systematic Review and Meta-Analysis
Source: Biomimetics (Basel). 2023 Jan 6;8(1):23. doi: 10.3390/biomimetics8010023 (PMC9844412; doi:10.3390/biomimetics8010023)
Supplement: Supplementary file 1 [file biomimetics-08-00023-s001.zip › Supplement-TableS2-Limeback_DH_Review-Search-details.pdf]

Supplement Table S2: Search Results Details - Dentin Hypersensitivity and Hydroxyapatite

Search words (up to May 1, 2022)	PubMed Result		SCOPUS Result (all fields)		EMBASE Result		Web of Science Result	
	Number of titles generated	Number of titles retrieved	Number of titles generated	Number of titles retrieved	Number of titles generated	Number of titles retrieved	Number of titles generated	Number of titles retrieved
apatite	29,254		128,123				33,946	
hydroxyapatite	34,403		239,333		41259		62,835	
nanohydroxyapatite	1,371		11,794				1,227	
nano-hydroxyapatite	1,249		23,679		1775		2,834	
hydroxyapatite + dentin(e)	1,188		12,675		680		1,408	
nano-hydroxyapatite + dentin(e)	57		1,650				74	
"hydroxylapatite"	19,011		26,910		3,775		4,280	
"hydroxylapatite" + dentin(e)	502		1,342		39		32	
sensitivity + dental	26,383		92,113		9,909		22,560	
"randomized clinical trial"	35,178		698,043		46,525		51,444	
"randomized clinical trial" + hydroxyapatite		50		4,995		39		81
"randomized clinical trial" + hydroxyapatite + sensitivity		11		591		8		10
"in vivo" + hydroxyapatite + sensitivity		123		7,578		67		53
"in vivo" + hydroxyapatite + sensitivity, dentin		10		901		7		4
sensitivity + dental + hydroxyapatite		264		4,513		124		127
(hyper)sensitivity + dentin(e) + hydroxyapatite		147		1,056		116		134

(hyper)sensitivity + nanohydroxyapatite		129		232		14		44
(hyper)sensitivity + dentin(e) + nanohydroxyapatite		35		175		12		38
toothpaste	6,132		21,748		10,398		5,123	
hydroxyapatite + toothpaste		174		2,322		232		232
hydroxyapatite + toothpaste + sensitivity		48		547		42		30
hydroxyapatite + toothpaste + hypersensitivity		37		600		38		62
nanohydroxyapatite + toothpaste + sensitivity		20		82		2		29
dentifrice	7,912		14,678		2,490		3,286	
hydroxyapatite + dentifrice		165		2,131		59		143
hydroxyapatite + dentifrice + sensitivity		43		558		11		21
hydroxyapatite + dentifrice + hypersensitivity		32		557		9		36
mouthwash	18,571		20,156		6,792		3,481	
hydroxyapatite + mouthwash		218		1,091		67		42
hydroxyapatite + mouthwash + sensitivity		30		220		6		2
hydroxyapatite + mouthwash + hypersensitivity		7		161		7		4
hydroxyapatite + gels(s)	3,801		68,720		1,626		5,192	
hydroxyapatite + gels(s) + sensitivity + dentin		241		606		1		9
nanohydroxyapatite + gel(s) + sensitivity		7		450		3		7
hydroxyapatite + gels(s) +		23		937		26		29

hypersensitivity								
hydroxyapatite + cream(s)		14		941		32		25
nanohydroxyapatite + cream(s)		3		89		5		2
hydroxyapatite + chewing gum		17		598		26		43
hydroxyapatite + chewing gum + sensitivity		2		111		4		0
Publications identified		1,850		32,042		957		1,207
Publications identified in total from primary databases	35,056							
Additional publications identified from other sources (Google Scholar; citation searching)	4,490							
Total relevant full publications included, retrieved and read in full after removing duplicates and irrelevant publications	44							

Supplement Table S2 shows the detailed results of the database search and the keywords used in that search. The rows shaded in grey indicate the quantitative results of titles retrieved using the search word combinations. Titles and abstracts were used to remove irrelevant publications for inclusion in the systematic review. The total is shown at the end of the table (44 publications were selected and their full texts extracted).
